# Highly Efficient Lithium Recovery from Pre-Synthesized Chlorine-Ion-Intercalated LiAl-Layered Double Hydroxides via a Mild Solution Chemistry Process

**DOI:** 10.3390/ma12121968

**Published:** 2019-06-19

**Authors:** Ying Sun, Rongping Yun, Yufeng Zang, Min Pu, Xu Xiang

**Affiliations:** State Key Laboratory of Chemical Resource Engineering, Beijing University of Chemical Technology, Beijing 100029, China; 2017210404@mail.buct.edu.cn (Y.S.); yuanrp@mail.buct.edu.cn (R.Y.); xiangxubuct@163.com (Y.Z.); pumin@mail.buct.edu.cn (M.P.)

**Keywords:** lithium recovery, LiAl-LDHs, reaction-coupled separation technology, salt lake brine

## Abstract

Lithium extraction from salt lake brine is critical for satisfying the increasing demand of a variety of lithium products. We report lithium recovery from pre-synthesized LiAl-layered double hydroxides (LDHs) via a mild solution reaction. Lithium ions were released from solid LiAl-LDHs to obtain a lithium-bearing solution. The LiAl-LDHs phase was gradually transformed into a predominantly Al(OH)_3_ phase with lithium recovery to the aqueous solution. The lithium recovery percentage and the concentration of the lithium-bearing solution were dependent on the crystallinity of LiAl-LDHs, the initial concentration of the LiAl-LDHs-1 slurry, the reaction temperature, and the reaction time. Under optimized conditions, the lithium recovery reached 86.2% and the Li^+^ concentration in the filtrate is 141.6 mg/L. Interestingly, no aluminum ions were detected in the filtrate after solid–liquid separation with high crystallinity LiAl-LDHs, which indicated the complete separation of lithium and aluminum in the liquid and solid phases, respectively. The ^27^Al NMR spectra of the solid products indicate that lithium recovery from the lattice vacancies of LiAl-LDHs affects the AlO_6_ coordination in an octahedral configuration of the ordered Al(OH)_3_ phase. The XPS O 1*s* spectra show that the O_ad_ peak intensity increased and the O_L_ peak intensity decreased with the increasing lithium recovery, which indicated that the Al-OH bond was gradually formed and the metal–oxygen–metal bond was broken.

## 1. Introduction

Lithium is the lightest metal with high electrochemical potential and is commonly used in lithium-ion batteries. Lithium-ion batteries are widely developed as efficient energy storage devices for electronic products and electric vehicles because they have a high energy density [1]. Lithium demand is expected to increase five-fold in the next decade [2]. Lithium-ion batteries account for 37% of the rechargeable batteries in the global market, and 39% of lithium resources are used to produce and manufacture batteries [3]. In 2019, global sales of electric vehicles (EVs) are likely to exceed 2.8 million which is expected to account for 3% of the total passenger car market, of which 68% will be battery electric vehicles (BEVs), and the rest will be plug-in hybrid electric vehicles (PHEVs) [4].

According to the US Geological Survey report in 2019, there are 62 million tons of lithium resources worldwide, 70% of which are found in salt lakes [5,6]. In China, there are 4.5 million tons of lithium resources, and 80% of them are contained in salt lake brine [7]. Therefore, extracting lithium from salt lake brine is an important way to obtain lithium resources. The great challenge is the high Mg/Li ratio in extracting lithium from salt lake brine in China. Currently, calcination, adsorption, extraction, and membrane technology are the available industrialized extraction methods. The calcination method is suitable for high-grade brines, but the extraction process consumes high energy and produces a large amount of HCl gas [8]. The adsorption method involves adsorbing Li^+^ selectively with adsorbent and then dissolving it in acid to separate Li^+^ from other ions. At present, manganese-based [9,10,11] and titanium-based [12,13] lithium-ion sieves have attracted the attention of researchers, and precursors include LiMnO_2_, Li_2_TiO_3_, among others. The extraction method involves selective extraction of lithium with organic solvents. The extractant with tributyl phosphate (TBP) in methyl isobutyl ketone (MIBK) and the co-extractant with FeCl_3_ have been extensively studied by researchers [14,15]. The membrane method produces ion separation by an electric field. A large number of studies on ion exchange membranes that can separate and concentrate ions to obtain lithium-rich brine have gradually been applied to the separation of metal ions [16,17].

Layered double hydroxides (LDHs) are a kind of intercalation material, and the chemical formula is [M^II^_1-x_ M^III^_x_(OH)_2_]^x+^[A^n-^]_x/n_·mH_2_O. A typical example is MgAl-layered double hydroxides (MgAl-LDHs), which have been widely used. The layer is formed by the uniform distribution of MgO_6_ and AlO_6_ octahedrons, possessing positive charge. The anions are intercalated into the interlayer, having supramolecular interactions with the layers to balance the positive charge of the layers. The methods for preparing LDHs have been studied. Lee et al. reported the preparation of LDHs by hydration of its corresponding metal oxides (MgO and Al_2_O_3_) [18]. The Mg^2+^ and Al(OH)_4_^-^ obtained by hydration of the metal oxide were incorporated into the MgAl-LDHs structure. In addition, the preparation methods of LDHs were reviewed by Conterosito et al. [19]. 

We recently reported a new reaction-coupled separation technology for extracting lithium from salt lake brine with a high Mg/Li ratio [20]. The layers of MgAl-LDHs consist of alternatively arranged MO_6_ (M = Mg or Al) octahedrons. Due to a large distortion angle, Li^+^ cannot enter the MgAl-LDHs structure. The Mg^2+^ and Li^+^ ions were separated efficiently, and MgAl-LDHs were prepared simultaneously. The mass ratio of Mg/Li in solution decreased significantly from 12.66 to less than 0.1, and the lithium loss rate was less than 10%. The chemical equations are shown in Equation (1) on the separation of magnesium and lithium from salt lake brine.

(1) $${6\mathrm{Mg}}^{2+}{+2\mathrm{AlCl}}_{3}{+16\mathrm{NaOH}+\mathrm{Na}}_{2}{\mathrm{CO}}_{3}{+\mathrm{mH}}_{2}{O\;=\;\mathrm{Mg}}_{6}{\mathrm{Al}}_{2}{\left(\mathrm{OH}\right)}_{16}{\mathrm{CO}}_{3}\cdot {\mathrm{mH}}_{2}{O+6\mathrm{Cl}}^{-}{+18\mathrm{Na}}^{+}$$

**Scheme 1:**
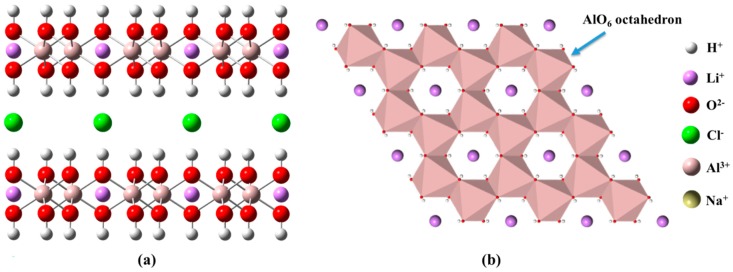
The structural model of chlorine-ion-intercalated LiAl-layered double hydroxides (LDHs) (**a**) front view, (**b**) top view.

LiAl-layered double hydroxides (LiAl-LDHs) are a unique type of hydrotalcite. The chemical name (IUPAC) is lithium aluminum chloride hydroxide hydrate. The general formula is [LiAl_2_(OH)_6_]X^n−^_1/n_·mH_2_O, in which X^n-^ is the anion, e.g., CO_3_^2-^, Cl^-^, NO^3^^-^. The Al(OH)_3_ with AlO_6_ octahedrons contained ordered vacancies. The lithium ions entered the vacancies, which made the layer positively charged. The anions were intercalated into the interlayer to keep charge balance [21,22,23]. The structural model of chlorine-ion-intercalated LiAl-LDHs is depicted in Scheme 1.

In this work, LiAl-LDHs were first synthesized from salt lake brine. The chemical composition of the brine is shown in Table 1. Subsequently, Li^+^ ions were recovered from the pre-synthesized LiAl-LDHs into an aqueous solution by mild chemical reaction. Equations (2) and (3) show the synthesis of chlorine-ion-intercalated LiAl-LDHs and the lithium recovery process. The two successive steps achieved the efficient extraction of lithium from salt lake brine. The effects of crystallinity of LiAl-LDHs, slurry concentration, reaction temperature, and reaction time on the lithium recovery percentage were discussed. Under the optimal conditions, the lithium recovery of LiAl-LDHs-1 prepared from the salt lake brine reached 86.2%, and the concentration of Li^+^ reached 141.6 mg/L. This study provides an alternative pathway to efficiently extract and recover lithium from salt lake brine.

(2) $${\mathrm{Li}}^{+}{+2\mathrm{AlCl}}_{3}{+6\mathrm{NaOH}+\mathrm{mH}}_{2}{O\;=\;\mathrm{LiAl}}_{2}{\left(\mathrm{OH}\right)}_{6}\mathrm{Cl}\cdot {\mathrm{mH}}_{2}{O+5\mathrm{Cl}}^{-}{+6\mathrm{Na}}^{+}$$

(3) $${\mathrm{LiAl}}_{2}{\left(\mathrm{OH}\right)}_{6}\mathrm{Cl}\cdot {\mathrm{mH}}_{2}{O\;=\;\mathrm{Li}}^{+}{+\mathrm{Cl}}^{-}{+2\mathrm{Al}(\mathrm{OH})}_{3}{+\mathrm{mH}}_{2}O$$

## 2. Materials and Methods 

### 2.1. Materials

AlCl_3_·6H_2_O was purchased from Aladdin Industrial Corporation (Shanghai China). NaOH was obtained from Beijing Chemical Corporation Ltd. (Beijing China).

### 2.2. Synthesis

The Mg^2+^ and Li^+^ ions are separated from salt lake brine by reaction-coupled separation technology to obtain the solution, i.e., the brine after separating Mg^2+^ ions [25]. The method for separating Mg^2+^ and Li^+^ is shown in the Supplementary Materials.

Solution A: AlCl_3_·6H_2_O had a concentration of 46.96 g/L was dissolved in the brine of 1 L after separating Mg^2+^ ions. The molar concentration of the relationship was [Li^+^]:[Al^3+^] = 1:2. By evaporation and concentration, varied initial Li^+^ concentrations were obtained. Solution B: NaOH was dissolved in deionized water to form a clear solution in which the [NaOH] = 2 mol/L. Solution B was dropped into solution A to keep the pH value at 7 during the reaction in the crystallization reactor. The deionized water used in the experiment was boiled and cooled to room temperature. Nitrogen gas was introduced into the reaction process to remove the influence of CO_3_^2-^ on the experimental results. The slurry was aged in a crystallization reactor at 80 °C for 12 h. The solid products obtained were LiAl-LDHs following filtration, washing, and drying.

LiAl-LDHs were dispersed in deionized water and heated in an oil bath at varied initial Li^+^ concentrations, slurry concentrations, recovery temperature, and recovery time. The solid product was recovered by filtration, washing, and drying, and the liquid phase was a lithium-bearing solution.

**Scheme 2:**
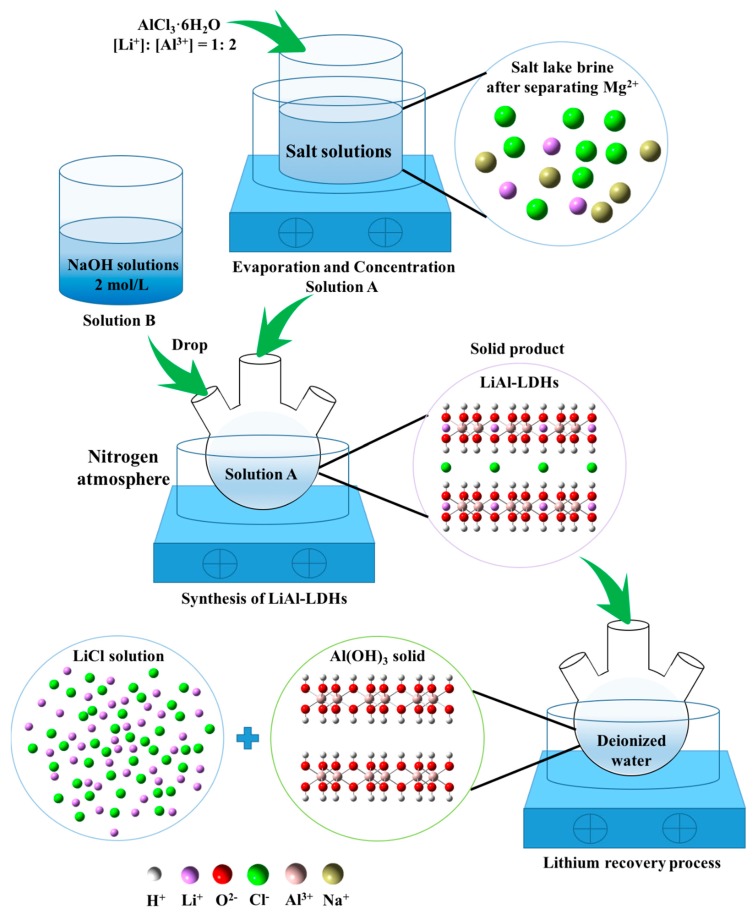
Flowchart of lithium recovery from pre-synthesized LiAl-layered double hydroxides.

The lithium recovery from pre-synthesized LiAl-layered double hydroxides is shown in Scheme 2.

#### 2.2.1. Crystallinity of LiAl-LDHs

By evaporation and concentration, the concentration of lithium ions in the brine after separating Mg^2+^ ions reached 6.75 g/L, 3.375 g/L, and 1.6875 g/L. Preparation of LiAl-LDHs under the above conditions was defined as LiAl-LDHs-1, LiAl-LDHs-2, and LiAl-LDHs-3, respectively. LiAl-LDHs were dispersed in water with a slurry concentration (LDHs (g)/water (L) ratio) of 10 g/L. The temperature and time of the thermal reaction were 85 °C and 90 min, respectively.

#### 2.2.2. Slurry Concentration of LiAl-LDHs-1

LiAl-LDHs-1 were dispersed in water at slurry concentrations of 10, 20, 30, or 50 g/L at 85 °C for 90 min. 

#### 2.2.3. Lithium Recovery Temperature

LiAl-LDHs-1 were dispersed in water at a slurry concentration of 10 g/L. The temperature of the thermal reaction was 65, 75, 85, or 95 °C. The reaction time was 90 min.

#### 2.2.4. Lithium Recovery Time

LiAl-LDHs-1 were dispersed in water at a slurry concentration of 10 g/L at 85 °C. The reaction time was set at 30, 60, 90, or 120 min.

### 2.3. Analysis

The X-ray diffraction patterns were obtained from XRD-6000 diffractometer (Shimadzu, Kyoto, Japan) in which the radiation was Cu Kα radiation at a sweep speed of 10°/min from 3° to 70°.

The Cl and C elements mass percentage were determined by ion chromatography (ICS-5000, ThermoFisher Scientific, Waltham, MA, USA) and elemental analysis (vario EL CUBE, elementar Analysensysteme GmbH, Langenselbold, Germany).

The solid sample was dispersed with ethanol and dripped onto a carbon-coated Cu grid, and the morphology of the sample was observed using transmission electron microscopy (HT7700, Hitachi, Tokyo, Japan).

Solid-state nuclear magnetic resonance examined the coordination information of ^27^Al and ^7^Li using NMR equipment (AV300, Bruker BioSpin GmbH, Rheinstetten, Germany). 

The composition and chemical structure of LiAl-LDHs and solid products after lithium recovery were characterized by X-ray photoelectron spectroscopy (ESCALAB 250, ThermoFisher Scientific, Waltham, MA, USA).

The concentration of the metal ion to be measured in solid and liquid phase products was diluted to 0–100 ppm to detect lithium ions and aluminum ions by inductively coupled plasma mass spectrometry (ICPS-7500 from Shimazduo, Kyoto, Japan). The formulas for Li^+^ concentration in the filtrate, lithium recovery percentage, and Al^3+^ dissolution percentage are shown in the Supplementary Materials Calculation Section.

## 3. Results and Discussion

**Figure 1:**
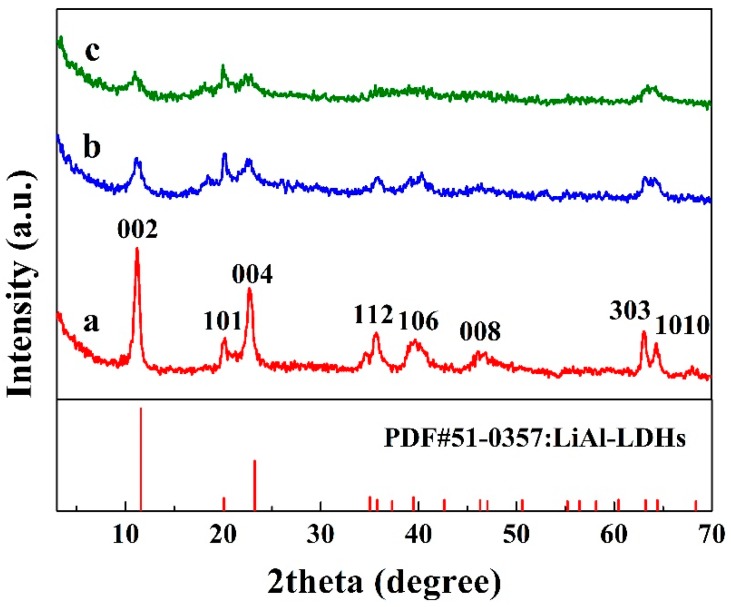
XRD patterns of LiAl-LDHs prepared from brine at varied initial Li^+^ concentrations (**a**) 6.75 g/L, (**b**) 3.375 g/L, (**c**) 1.6875 g/L.

**Figure 2:**
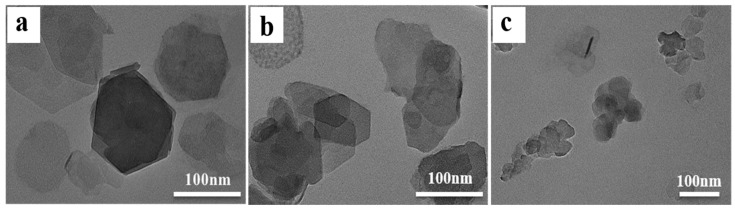
TEM micrographs of LiAl-LDHs at varied initial Li^+^ concentrations (**a**) 6.75 g/L, (**b**) 3.375 g/L, (**c**) 1.6875 g/L.

LiAl-LDHs with varied initial Li^+^ concentrations were prepared from salt lake brine. The chemical compositions of the prepared LiAl-LDHs are listed in Table 2. The Li/Al molar ratio of LiAl-LDHs-1, LiAl-LDHs-2, and LiAl-LDHs-3 were 1:2.00, 1:2.02, and 1:2.14, respectively, which are consistent with stoichiometry. The mass percentage of Cl element was 14.05%, 14.02%, and 13.89%, and the Li/Cl molar ratio was 1.00. The Cl/C molar ratio of LiAl-LDHs-1, LiAl-LDHs-2, and LiAl-LDHs-3 were 9.90, 9.88, and 9.80, respectively (Table 2), which indicated that the amount of CO_3_^2-^ in LiAl-LDHs was negligible. The XRD pattern (Figure 1) demonstrated that the diffraction line was consistent with planes (002), (101), (004), (112), (106), (008), (303), and (1010) of [LiAl_2_(OH)_6_]Cl·xH_2_O (JCPDS Card No. 51-0357) [25,26]. The chlorine-ion-intercalated LiAl-layered double hydroxides were prepared via a mild solution chemistry process from salt lake brine. The higher the lithium ion concentration, the sharper the diffraction peak, which indicated that the crystallinity of LiAl-LDHs-1, LiAl-LDHs-2, and LiAl-LDHs-3 decreased in turn. As shown in Figure 2, the crystal growth of LiAl-LDHs was complete and displayed a hexagonal sheet morphology, which is characteristic of LDHs, and the crystallization is consistent with the XRD pattern [27,28]. The ^27^Al NMR spectra (Figure S1) of LiAl-LDHs-1 revealed a chemical shift of ^27^Al as 6.39 ppm; that is, the Al environment was six-coordinated Al [29]. This result was consistent with researchers’ understanding of LiAl-LDHs [26]. The XPS O 1*s* spectra of LiAl-LDHs-1 (Figure S2) were divided into three peaks with binding energies of 532.5, 531.9, and 531.3 eV. These binding energies were attributed to H_2_O(O_w_), OH^-^(O_ad_), and O^2-^(O_L_), respectively [30].

### 3.1. Crystallinity of LiAl-LDHs

**Figure 3:**
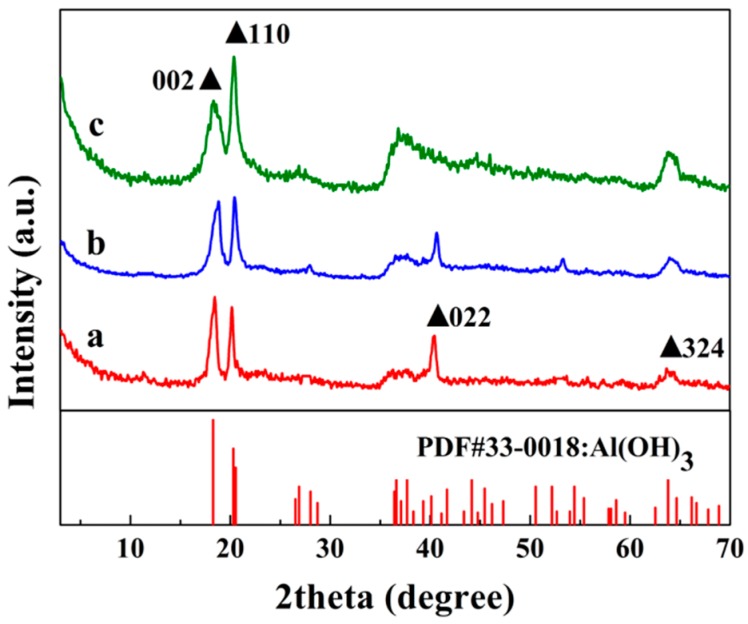
XRD patterns of solid products after lithium recovery from LiAl-LDHs at varied initial Li^+^ concentrations (**a**) 6.75 g/L, (**b**) 3.375 g/L, (**c**) 1.6875 g/L (▲ Al(OH)_3_).

The effect of the thermal reaction of LiAl-LDHs with different crystallinity on lithium recovery was studied. Figure 3 shows the XRD pattern of solid products after lithium recovery by the thermal reaction. The position of the diffraction peak of the thermally reacted solid product of LiAl-LDHs was consistent with Al(OH)_3_ as a gibbsite phase (JCPDS Card No. 33-0018) [31], indicating that Li^+^ was released from the solid product. The diffraction peaks are different at 2theta 36° and 63° in Figure 3, as such they cannot be used as evidence for identifying substances because they are weaker diffraction signals. In order to further discuss the relationship between lithium recovery and crystallinity, we studied the changes in Li+ concentration in the filtrate, the chemical shift of ^27^Al, and binding energy.

The concentration of Li^+^ in the filtrate decreased with the decreasing of crystallinity because the amount of Li^+^ extracted decreased from LiAl-LDHs (Table 3). Because of the low crystallinity, the distortion of AlO_6_ octahedrons occurred. The Al-O chemical bonds were broken under the mild chemistry process, allowing a small amount of Al^3+^ to be dissolved in the solution. 

The weight of LiAl-LDHs is 1 g. The volume of the solution after the experiment is 167 mL, 143 mL, and 142 mL for LiAl-LDHs-1, LiAl-LDHs-2, and LiAl-LDHs-3, respectively.

**Figure 4:**
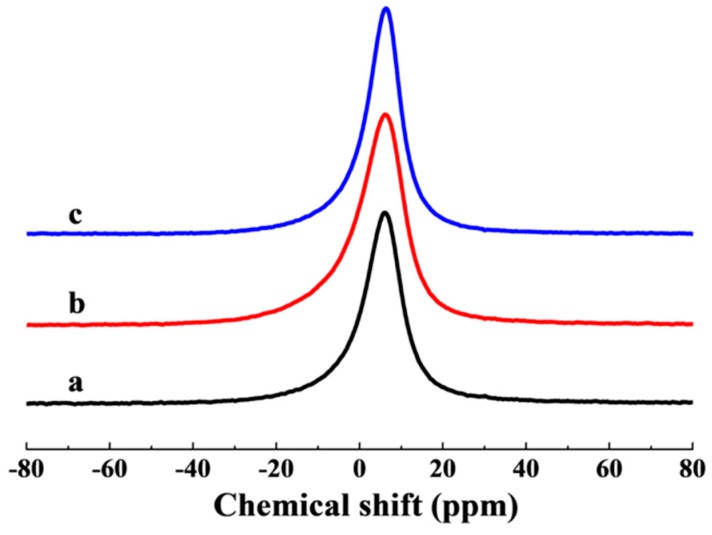
^27^Al NMR spectra of the solid products after lithium recovery from LiAl-LDHs at varied initial Li^+^ concentrations (**a**) 6.75 g/L, (**b**) 3.375 g/L, (**c**) 1.6875 g/L.

The dissolution of Li^+^ with Cl^-^ from LiAl-LDHs into aqueous solution changes the Al-O coordination environment of solid products after lithium recovery. The recovery rate of lithium from LiAl-LDHs-1 reached 86.2% to deform the Al-O octahedron [32], and the chemical shift of ^27^Al was shifted from 6.39 ppm (^27^Al chemical shift of LiAl-LDHs-1, Figure S1) to the low-field by 0.32 ppm (Figure 4). Moreover, the chemical shifts of LiAl-LDHs-2 and LiAl-LDHs-3 were shifted to the low-field by 0.24 and 0.09 ppm, respectively. The chemical shift of ^27^Al was shifted towards the low-field with increasing crystallinity of LiAl-LDHs. Larger lithium recovery percentage lead to a greater impact on aluminum coordination. 

**Figure 5:**
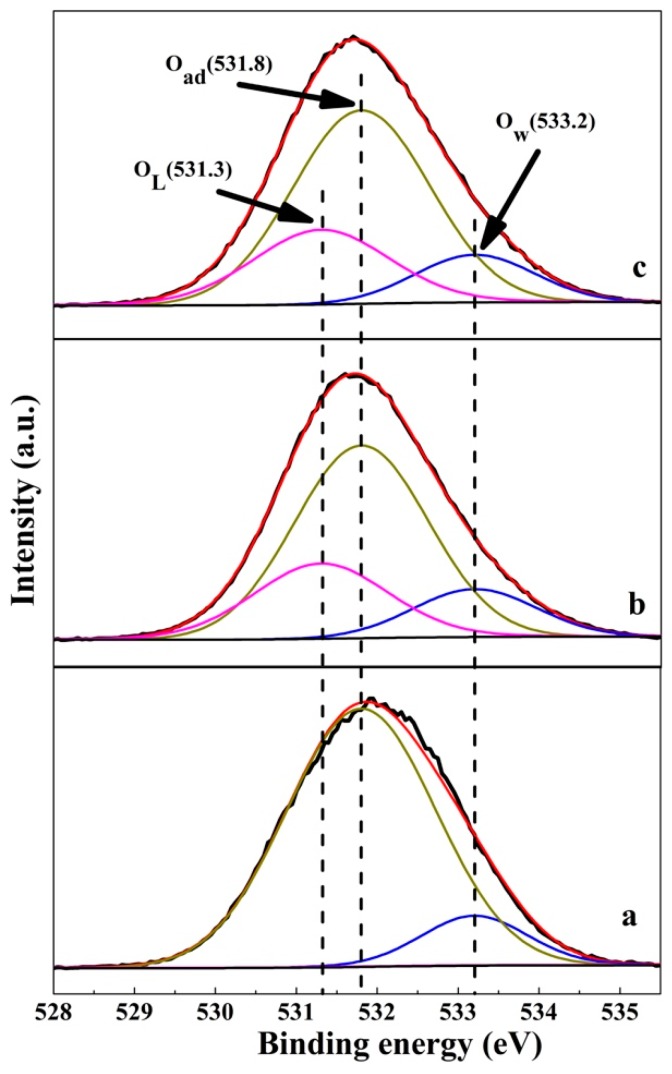
XPS O 1*s* spectra of the solid products after lithium recovery from LiAl-LDHs at varied initial Li^+^ concentrations (**a**) 6.75 g/L, (**b**) 3.375 g/L, (**c**) 1.6875 g/L.

As the thermal reaction proceeded, the binding energy of O 1*s* changed due to the conversion of solids from LiAl-LDHs to Al(OH)_3_. The XPS O 1*s* spectra of the solid products after lithium recovery (Figure 5) was divided into three peaks with binding energies of 533.2(O_W_), 531.8(O_ad_), and 531.3 eV(O_L_) [33]. Since the O_w_ intensity was greatly affected by the drying condition, it will not be discussed here. Compared with LiAl-LDHs-1 (Figure S2), the O_ad_ peak intensity of the solid product after lithium recovery increased, and the O_L_ peak intensity decreased, which meant that the metal hydroxides or the hydroxyl group gradually increased and the metal–oxygen–metal bond gradually broke. This result is consistent with the process of forming Al(OH)_3_ from LiAl-LDHs. In Figure 5, the solid product after lithium recovery tends to form Al(OH)_3_ when the crystallinity degree of LiAl-LDHs increased.

### 3.2. Slurry Concentration of LiAl-LDHs-1

Lithium ions were extracted from LiAl-LDHs-1 at different slurry concentrations ranging from 10 g/L to 50 g/L. At 10 g/L, the diffraction line can be indexed to the planes (002), (110), (022), and (324) of Al(OH)_3_ (JCPDS Card No. 33-0018) in the XRD patterns [34,35]. As the slurry concentration increased, the dissolution of Li^+^ from the LiAl-LDHs-1 became more incomplete, as the LiAl-LDHs phase existed at 2theta 11.59°, 23.21°, 35.74°, 63.20°, and 64.43° (Figure S3).

**Figure 6:**
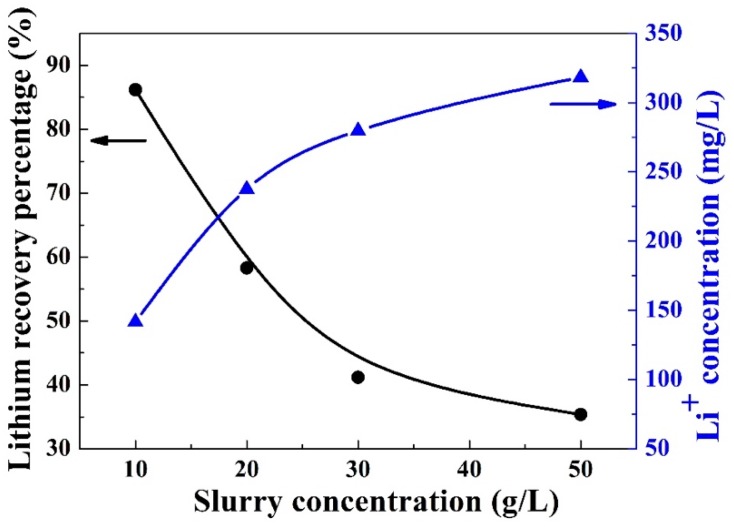
Lithium recovery percentage (round symbol) and Li^+^ concentration in the filtrate (triangle symbol) from LiAl-LDHs-1 slurry at varying concentrations of 10 g/L, 20 g/L, 30 g/L, 50 g/L.

Figure 6 shows the effects of slurry concentration on the lithium recovery percentage and Li^+^ concentration in the filtrate, which is consistent with XRD data. With increasing slurry concentration, the lithium recovery percentage decreased, on the contrary, Li^+^ concentration in the filtrate increased gradually. The lithium recovery percentage was 86.2%, and the concentration of Li^+^ in the liquid phase was 141.6 mg/L when the slurry concentration was 10 g/L. Continuing to increase the amount of reactants (50 g/L) caused the lithium recovery percentage to be only 35.4%, but the Li^+^ concentration in the filtrate reached 318.3 mg/L. According to Equation (3), the increase of product concentration will affect the balance of reaction, and the amount of lithium dissolved from the solid phase decreases, thus the lithium recovery percentage decreased. Al^3+^ was not detected in the detection limit by ICP, so the lithium ion solution without impure ions was obtained, which led to the extraction and enrichment of lithium from salt lake brine.

The chemical shift of ^27^Al varies with the slurry concentration according to NMR. The chemical shift was 6.07, 6.30, 6.32, and 6.38 ppm at 10 g/L, 20 g/L, 30 g/L, and 50 g/L, respectively (Figure S4) [36]. This change was affected by the lithium recovery percentage, which was the same trend as shown in Figure 4. The chemical shift was clearly more biased toward the low-field with the increase in lithium recovery percentage (i.e., decreased slurry concentration), which meant that the LiAl-LDHs phase converted more to the Al(OH)_3_ phase. 

### 3.3. Lithium Recovery Temperature

Lithium-ion extraction at different reaction temperatures was investigated. With increasing reaction temperature, the LiAl-LDHs phase gradually disappeared, showing the Al(OH)_3_ phase (Figure S5). No other phase was detected at 85 °C and 95 °C.

**Figure 7:**
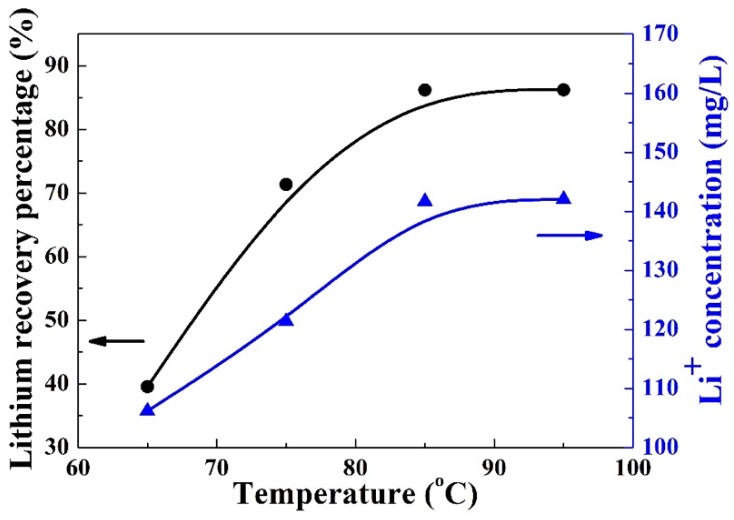
Lithium recovery percentage (round symbol) and Li^+^ concentration in the filtrate (triangle symbol) from LiAl-LDHs-1 at varied recovery temperatures of 65 °C, 75 °C, 85 °C, 95 °C.

The temperature dependence of the lithium recovery percentage was investigated over the range of 65 °C to 95 °C (Figure 7). At lower temperatures (65–75 °C), the lithium recovery percentage is more sensitive to temperature, so the lithium recovery percentage increased rapidly as the temperature rose. At 95 °C, the chemical reaction reached equilibrium, that is, the amount of lithium dissolved from the solid phase was balanced with the amount of lithium entering the vacancy of Al(OH)_3_. The lithium recovery percentage and Li^+^ concentration in the filtrate were no longer increased.

In the NMR spectra (Figure S6A), the peaks corresponding to the chemical shift of ^27^Al in the solid product after lithium recovery were 6.35, 6.19, and 6.07 ppm at 65 °C, 75 °C, and 85 °C, respectively. The main peak of the ^27^Al NMR spectra was 6 ppm at 95 °C, and a peak appeared with a relatively broad low-frequency shoulder at 1.29 ppm (Figure S6B) [37]. This phenomenon was consistent with the literature where Al(OH)_3_ as gibbsite phase had a shoulder peak [38]. With increasing temperature, the solid product was transformed to the Al(OH)_3_ phase, where the amount of Li^+^ dissolved with Cl^-^ from LiAl-LDHs-1 increased; in other words, the lithium recovery percentage increased. Through the lithium recovery percentage, the requirements of lithium recovery could be met at a temperature of 85 °C and 95 °C. Obviously, nearly the same effect was achieved at 95 °C and reduced energy consumption was achieved at 85 °C.

### 3.4. Lithium Recovery Time

**Figure 8:**
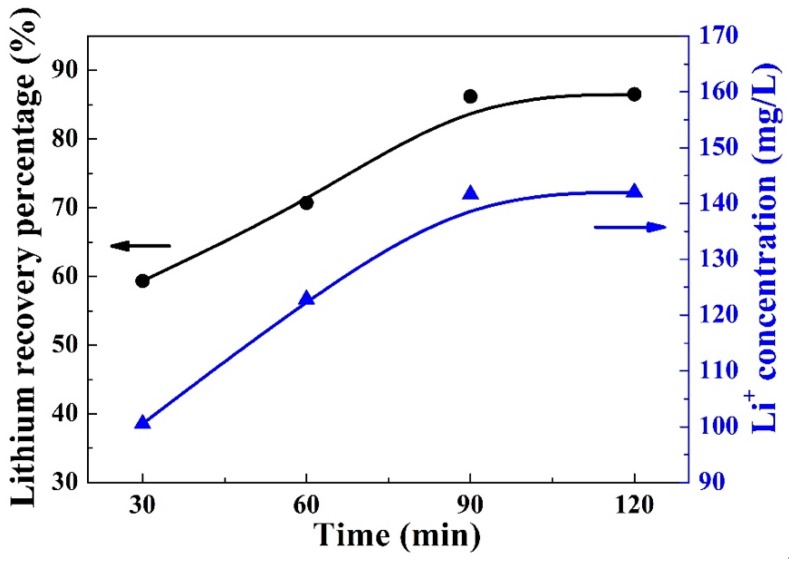
Lithium recovery percentage (round symbol) and Li^+^ concentration in the filtrate (triangle symbol) from LiAl-LDHs-1 at varied recovery times of 30 min, 60 min, 90 min, 120 min.

The reaction time determines the degree of the reaction. The XRD patterns (Figure S7) show that the LiAl-LDHs phase gradually disappears with increasing reaction time from 30 min to 120 min. After 90 min of reaction, a sharp and high-intensity Al(OH)_3_ characteristic diffraction peak appeared with planes of (002), (110), (022), and (324). The effect of time on the lithium recovery percentage was consistent with the temperature, as lithium recovery percentage increases with increasing reaction time. The reaction was essentially complete after 90 min because the lithium recovery percentage no longer increased (Figure 8). The Li^+^ concentration was 141.6 mg/L and 142.0 mg/L in the liquid phase at 90 min and 120 min, respectively. No Al^3+^ dissolution was observed within the detection limit. Within the range of error, we consider that the Li^+^ concentration in the filtrate is the same with reaction times of 90 min and 120 min.

We repeated the experiments and analyzed the data under the varying concentrations, recovery temperatures, and recovery times (Tables S1–S3). The results are repeatable.

## 4. Conclusions

Lithium was extracted from LiAl-LDHs prepared from salt lake brine using reaction-coupled separation technology. Lithium was released from the lattice vacancies of the ordered AlO_6_ octahedrons in pre-synthesized LiAl-Cl-LDHs. Lithium and aluminum were effectively separated to obtain a pure lithium salt solution, which can be used in industrial production. As the crystallinity of LiAl-LDHs decreased, the lithium recovery percentage decreased. Al^3+^ was detected to be dissolved in the liquid phase due to the Al-O bonds being more easily broken during the thermal reaction with low crystallinity LiAl-LDHs. The slurry concentration had an opposite effect on the lithium recovery percentage and the Li^+^ concentration in the filtrate. The lithium recovery percentage decreased as the slurry concentration increased because the lithium-ion extraction rate was suppressed. The dissolution of Li^+^ from LiAl-LDHs affected the chemical environment of aluminum, where the ^27^Al chemical peak shifted to the low-field and a peak with a broad low-frequency shoulder appeared. The Al-OH bond gradually formed and the metal-oxygen-metal bond was gradually broken with the increase of lithium recovery percentage, and the solid phase product was converted to Al(OH)_3_ when LiAl-LDHs disappeared. When the slurry concentration was 10 g/L, the lithium recovery percentage of LiAl-LDHs-1 was 86.2% at 85 °C for 90 min, and the Li^+^ concentration in the filtrate was 141.6 mg/L. Al^3+^ was hardly detected in the solution under various reaction conditions. This work provides a method for extracting lithium ions from salt lake brine and for obtaining a lithium-bearing solution, which is capable of directly producing lithium salts, such as Li_2_CO_3_ and LiOH.

## Figures and Tables

**Table 1:** The chemical composition of the brine after separating Mg2+ ions [24].

	Mg^2+^	Li^+^	K^+^	Na^+^	B_2_O_3_	Cl^-^	SO_4_^2-^	CO_3_^2-^
Concentration (g/L)	0.054	0.675	0.84	32.9	1.73	42.87	3.29	8.57

**Table 2:** The chemical compositions of the prepared LiAl-LDHs.

	LiAl-LDHs-1	LiAl-LDHs-2	LiAl-LDHs-3
Li (wt%)	2.75	2.74	2.72
Al (wt%)	21.37	21.53	22.63
Cl (wt%)	14.05	14.02	13.89
C (wt%)	0.48	0.48	0.47
Li/Al molar ratio	1:2.00	1:2.02	1:2.14
Cl/C molar ratio	9.91	9.89	9.80
Li/Cl molar ratio	1.00	1.00	1.00

**Table 3:** Analyses on the solution after lithium recovery from LiAl-LDHs at varied initial Li^+^ concentrations.

Sample	Lithium Recovery Percentage (%)	Li^+^ Concentration in Filtrate (mg/L)	Al^3+^ Dissolution Percentage (%)
LiAl-LDHs-1	86.2	141.6	-
LiAl-LDHs-2	66.6	127.4	-
LiAl-LDHs-3	56.2	107.4	0.32

## Supplementary Materials

The following are available online at https://www.mdpi.com/1996-1944/12/12/1968/s1, the content of elements in LiAl-LDHs; additional NMR spectra, XPS O 1*s* spectra, XRD patterns.
