# Supplementary material for: Highly Efficient Lithium Recovery from Pre-Synthesized Chlorine-Ion-Intercalated LiAl-Layered Double Hydroxides via a Mild Solution Chemistry Process
Source: Materials (Basel). 2019 Jun 19;12(12):1968. doi: 10.3390/ma12121968 (PMC6630303; doi:10.3390/ma12121968)
Supplement: Supplementary file 1 [file materials-12-01968-s001.pdf]

**Supplementary Materials**

# Highly Efficient Lithium Recovery from Pre-Synthesized Chlorine-Ion-Intercalated LiAl-Layered Double Hydroxides via a Mild Solution Chemistry Process

Ying Sun, Rongping Yun, Yufeng Zang, Min Pu, Xu Xiang*

State Key Laboratory of Chemical Resource Engineering, Beijing University of Chemical Technology, Beijing 100029, China; Email: xiangxu@mail.buct.edu.cn

**Experimental Section**

**Calculation Section**

**Table Captions**

**Table S1.** The results of repeated experiments under varied concentrations.
**Table S2.** The results of repeated experiments under varied recovery temperatures.
**Table S3.** The results of repeated experiments under varied recovery times.

**Figure Captions**

**Figure S1.** 27Al NMR spectra of LiAl-LDHs-1 sample.
**Figure S2.** XPS O 1s spectra of LiAl-LDHs-1 sample.
**Figure S3.** XRD patterns of solid products after lithium recovery from LiAl-LDHs-1 slurry at varied concentration of (a) 10 g/L, (b) 20 g/L, (c) 30 g/L, (d) 50 g/L (▲ Al(OH)3; ◆ LiAl-LDHs).
**Figure S4.** 27Al NMR spectra of the solid products after lithium recovery from LiAl-LDHs-1 slurry at varied concentration.
**Figure S5.** XRD patterns of solid products after lithium recovery from LiAl-LDHs-1 at varied recovery temperature of (a) 65 °C, (b) 75 °C, (c) 85 °C, (d) 95 °C (▲ Al(OH)3; ◆ LiAl-LDHs).
**Figure S6.** 27Al NMR spectra of the solid products after lithium recovery from LiAl-LDHs-1 at (A) varied recovery temperature, (B) 95 °C.
**Figure S7.** XRD patterns of solid products after lithium recovery from LiAl-LDHs-1 at varied recovery time of (a) 30 min, (b) 60 min, (c) 90 min, (d) 120 min (▲ Al(OH)3; ◆ LiAl-LDHs).

## Experimental Section

The $Mg^{2+}$ and $Li^{+}$ ions are separated from salt lake brine by reaction-coupled separation technology. Firstly, the brine of 50 mL is diluted to 150 mL. The $AlCl_3{\cdot}6H_2O$ was dissolved in the brine to form salt solution ($Mg^{2+}/Al^{3+}$=3.0). NaOH and $Na_2CO_3$ were dissolved in deionized water to form base solution, in which [NaOH] = 1.6[$Mg^{2+}$+$Al^{3+}$] and [$CO_3^{2-}$] = 2.0[$Al^{3+}$]. Secondly, the salt solution and base solution were simultaneously poured into a colloid mill at a rotating speed of 4000 rpm for rapid nucleation within several minutes. The slurry was transferred to a crystallization reactor and aged at 100 °C for 6 h. Finally, the suspension was filtered for solid-liquid separation.

## Calculation Section

The Li+ concentration in the filtrate (equation (S1)), lithium recovery percentage (equation (S2)) and Al3+ dissolution percentage (equation (4S)) were then calculated. The solid LDHs were dissolved in a nitric acid solution, and the liquid phase product was directly measured after dilution.

$$C=c(Li^{+})\cdot F, \quad (S1)$$

where C refers to the Li+ concentration in the filtrate, c(Li+) is the concentration of Li+ measured in the filtrate, and F is the diluting factor of the ICP test.

$$R=C\cdot V/(p\cdot m), \quad (S2)$$

where R refers to the lithium recovery percentage, V is the filtrate volume after the reaction, m is the mass of the LDHs used for the reaction, and p is the mass percentage of lithium in LDHs.

$$p=(c(Li^{+})_{LDH}\cdot V_{LDH})/m_{LDHs}, \quad (S3)$$

where c(Li+)LDH is the concentration of Li+ measured in LDHs, VLDH is the nitric acid solution volume for testing, and mLDHs is the mass of the LDHs used for testing.

$$R_{Al}=c(Al^{3+})\cdot F\cdot V/(p_{Al}\cdot m), \quad (S4)$$

where RAl refers to the Al3+ dissolution percentage, c(Al3+) is the concentration of Al3+ measured in the filtrate, and pAl is the mass percentage of aluminum in LDHs.

$$p_{Al}=(c(Al^{3+})_{LDH}\cdot V_{LDH})/m_{LDHs}, \quad (S5)$$

where c(Al3+)LDH is the concentration of Al3+ measured in LDHs.

**Table S1.** The results of repeated experiments under varied slurry concentrations.

| Slurry concentrations (g/L) | 10 | 20 | 30 | 50 |
|---|---|---|---|---|
| Lithium recovery percentage (%)[1] | 86.2 | 58.3 | 41.2 | 35.4 |
| Lithium recovery percentage (%)[2] | 86.2 | 58.4 | 41.1 | 35.5 |
| Lithium recovery percentage (%)[3] | 86.3 | 58.4 | 41.2 | 35.4 |
| RMD (%)[4] | 0.05 | 0.08 | 0.11 | 0.13 |
| $Li^{+}$ concentration (mg/L)[1] | 141.6 | 237.5 | 279.7 | 318.3 |
| $Li^{+}$ concentration (mg/L)[2] | 141.6 | 237.3 | 280.0 | 318.5 |
| $Li^{+}$ concentration (mg/L)[3] | 141.4 | 237.8 | 279.7 | 318.0 |
| RMD (%)[4] | 0.06 | 0.07 | 0.05 | 0.06 |

[1] The first experiment
[2] The second experiment
[3] The third experiment
[4] Relative Mean Deviation

**Table S2. The results of repeated experiments under varied recovery temperatures.**

| Temperatures (°C) | 65 | 75 | 85 | 95 |
|---|---|---|---|---|
| Lithium recovery percentage (%)[1] | 39.6 | 71.4 | 86.2 | 86.2 |
| Lithium recovery percentage (%)[2] | 39.5 | 71.5 | 86.2 | 86.4 |
| Lithium recovery percentage (%)[3] | 39.6 | 71.5 | 86.3 | 86.3 |
| RMD (%)[4] | 0.11 | 0.06 | 0.05 | 0.08 |
| $Li^+$ concentration (mg/L)[1] | 106.2 | 121.3 | 141.6 | 142.0 |
| $Li^+$ concentration (mg/L)[2] | 106.1 | 121.5 | 141.6 | 142.2 |
| $Li^+$ concentration (mg/L)[3] | 105.9 | 121.5 | 141.4 | 142.2 |
| RMD (%)[4] | 0.10 | 0.07 | 0.06 | 0.06 |

[1] The first experiment
[2] The second experiment
[3] The third experiment
[4] Relative Mean Deviation

**Table S3. The results of repeated experiments under varied recovery times.**

| Time (min) | 30 | 60 | 90 | 120 |
|---|---|---|---|---|
| Lithium recovery percentage (%)[1] | 59.4 | 70.7 | 86.2 | 86.5 |
| Lithium recovery percentage (%)[2] | 59.3 | 70.8 | 86.2 | 86.3 |
| Lithium recovery percentage (%)[3] | 59.4 | 70.6 | 86.3 | 86.5 |
| RMD (%)[4] | 0.07 | 0.09 | 0.05 | 0.10 |
| $Li^+$ concentration (mg/L)[1] | 100.5 | 112.9 | 141.6 | 142.0 |
| $Li^+$ concentration | 100.4 | 113.0 | 141.6 | 142.0 |

| | | | | |
|---|---|---|---|---|
| (mg/L)[2] | | | | |
| $Li^+$ concentration (mg/L)[3] | 100.3 | 112.8 | 141.4 | 141.9 |
| RMD (%)[4] | 0.07 | 0.06 | 0.06 | 0.08 |

[1] The first experiment
[2] The second experiment
[3] The third experiment
[4] Relative Mean Deviation

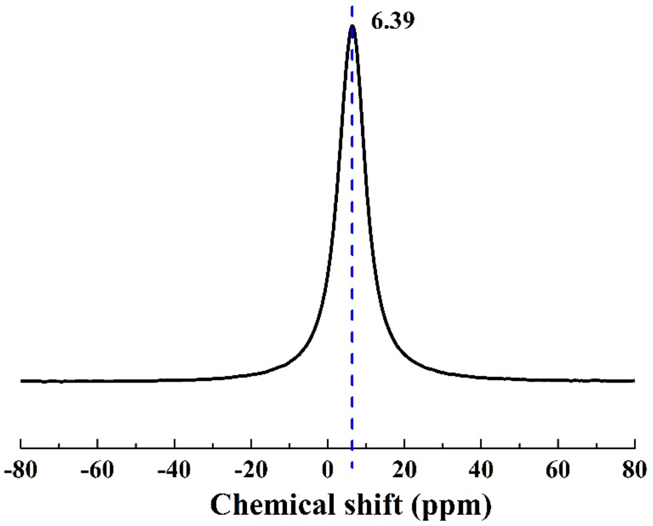


**Figure S1.** $^{27}Al$ NMR spectra of LiAl-LDHs-1 sample.

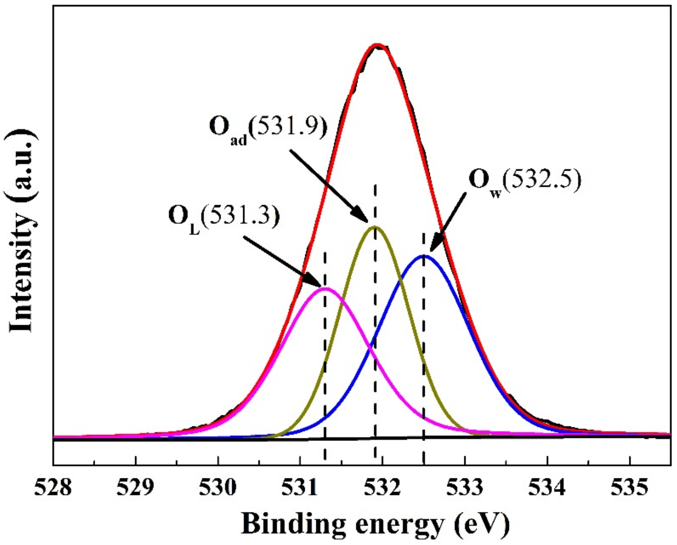


**Figure S2.** XPS O 1*s* spectra of LiAl-LDHs-1 sample.

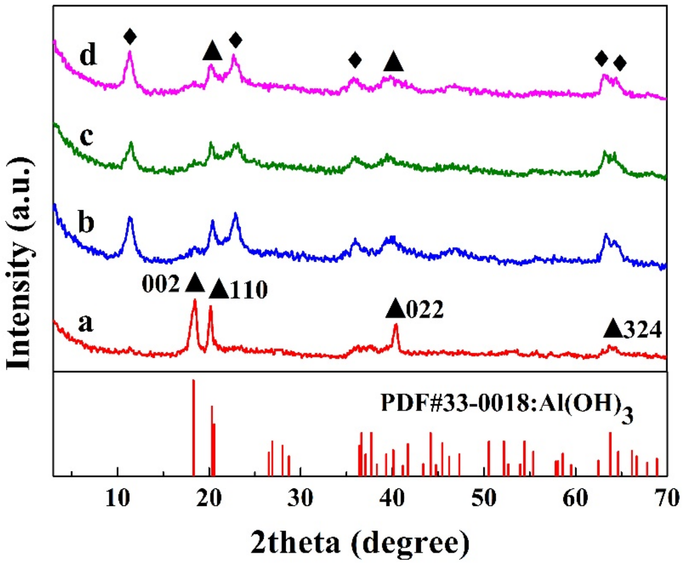


**Figure S3.** XRD patterns of solid products after lithium recovery from LiAl-LDHs-1 slurry at varied concentration of (a) 10 g/L, (b) 20 g/L, (c) 30 g/L, (d) 50 g/L

(▲ $Al(OH)_3$; ◆ LiAl-LDHs).

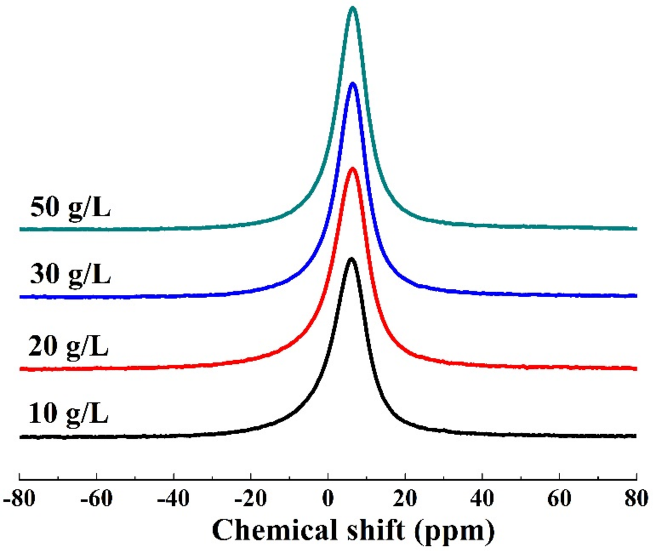


**Figure S4.** 27Al NMR spectra of the solid products after lithium recovery from LiAl-LDHs-1 slurry at varied concentration.

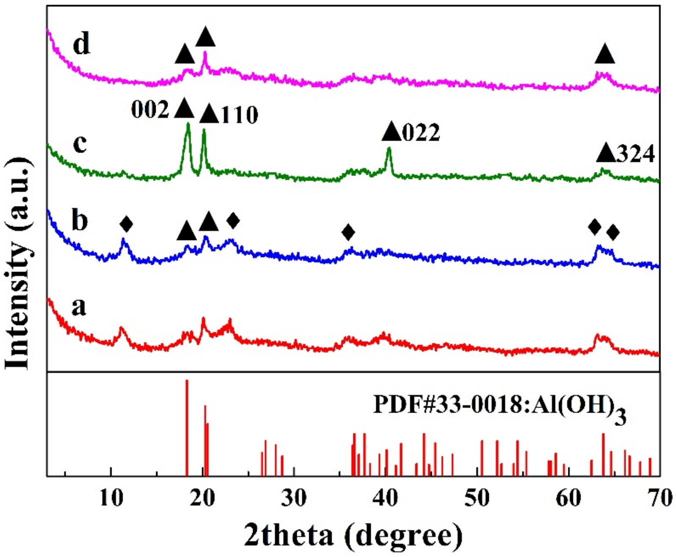


**Figure S5.** XRD patterns of solid products after lithium recovery from LiAl-LDHs-1 at varied recovery temperature of (a) 65 °C, (b) 75 °C, (c) 85 °C, (d) 95 °C

(▲ $Al(OH)_3$; ◆ LiAl-LDHs).

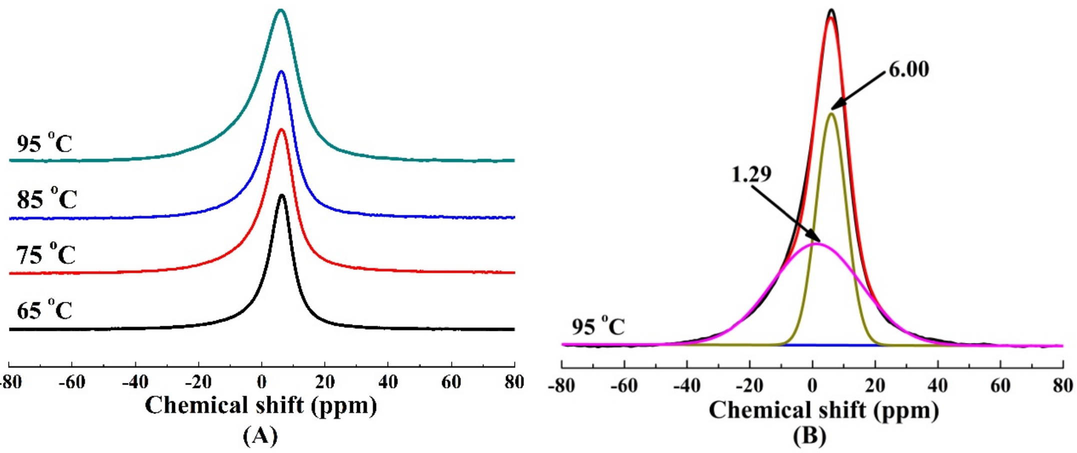


**Figure S6.** 27Al NMR spectra of the solid products after lithium recovery from LiAl-LDHs-1 (A) at varied recovery temperature, (B) at 95 °C.

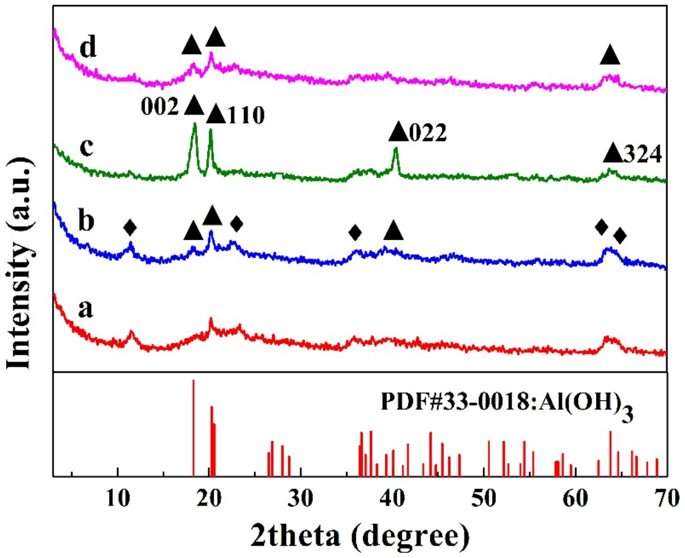


**Figure S7.** XRD patterns of solid products after lithium recovery from LiAl-LDHs-1 at varied recovery time of (a) 30 min, (b) 60 min, (c) 90 min, (d) 120 min

(▲ $Al(OH)_3$; ◆ LiAl-LDHs).
